# Effects of *Wolbachia* removal on microbial composition and diversity in *Aedes albopictus*: implication of using *w*AlbB for discriminating irradiation-based sterile and wild males

**DOI:** 10.1186/s40249-025-01343-3

**Published:** 2025-07-14

**Authors:** Paerhande Dilinuer, Ming Li, Datao Lin, Yu Wu, Zhongdao Wu, Xiaoying Zheng, Dongjing Zhang

**Affiliations:** 1 Chinese Atomic Energy Agency Center of Excellence On Nuclear Technology Applications for Insect Control, Key Laboratory of Tropical Disease Control of the Ministry of Education, Sun Yat-Sen University, Guangzhou, 510080 China; 2 International Atomic Energy Agency Collaborating Centre, Sun Yat-Sen University, Guangzhou, 510080 China; 3 Guangdong Provincial Engineering Technology Research Center for Diseases-Vectors Control, Sun Yat-Sen University, Guangzhou, 510080 China

**Keywords:** Sterile insect technique, Bacterial diversity, Bacterial composition, *Wolbachia w*AlbB, *Enterococcus*

## Abstract

**Background:**

The sterile insect technique (SIT) requires distinguishing sterile from wild male mosquitoes to evaluate male qualities and maintain an appropriate release ratio for efficient population suppression. Current dye/powder marking methods have limitations and may affect SIT effectiveness, necessitating alternative discrimination strategies. *Aedes albopictus* naturally harbors two *Wolbachia* infections (*w*AlbA/*w*AlbB), which can be eliminated via tetracycline. Although *Wolbachia* removal minimally affect host fitness, its impact on microbiota remains unclear. Characterizing post-elimination microbial communities is the first step to identify novel endogenous biomarkers for SIT monitoring.

**Methods:**

We analyzed the bacterial diversity and composition of two strains of wild-type GUA (*Wolbachia*-infected) and GT (*Wolbachia*-free) mosquitoes using the *16S r*RNA V3-V4 region sequencing. qPCR was employed to confirm the relative abundance of four major bacterial genera, while PCR was used to validate selected biomarkers for distinguishing factory-reared sterile males from wild males. Kruskal-Wallis or Mann-Whitney test was used to analyze the comparable parameters between GUA and GT strains.

**Results:**

Five-day-old GUA and GT females showed similar microbial diversity/composition, while young males shared diversity but differed in composition. The core microbiota in both strains consisted of Proteobacteria (64.27%), Firmicutes (16.09%), Actinobacteriota (11.22%), and Bacteroidota (4.96%). *Asaia* was dominant in both strains (GUA: 47.33%; GT: 32.69%), whereas *Enterococcus* increased in GT males with aging. *Wolbachia* was absent in GT mosquitoes, and *Elizabethkingia* was undetected in GUA males. qPCR further confirmed these trends. PCR analysis revealed that *w*AlbB exhibited higher stability in differentiating factory-reared GT males from their wild counterparts (96.7% infection in field males, *n* = 60) compared to *w*AlbA (61.7%, *n* = 60) or *Enterococcus* (65.8%, *n* = 120). The mark-release-recapture experiment further confirmed the detectability using *w*AlbB biomarker.

**Conclusions:**

Without obvious fitness costs observed previously in the *Ae. albopictus* GT strain compared to GUA strain, the removal of *Wolbachia* significantly changes the microbial composition in male mosquitoes in this study*. Wolbachia w*AlbB is recommended as a reliable biomarker for distinguishing sterile males from wild males when using GT strain in SIT programs targeting *Ae. albopictus*.

**Graphical Abstract:**

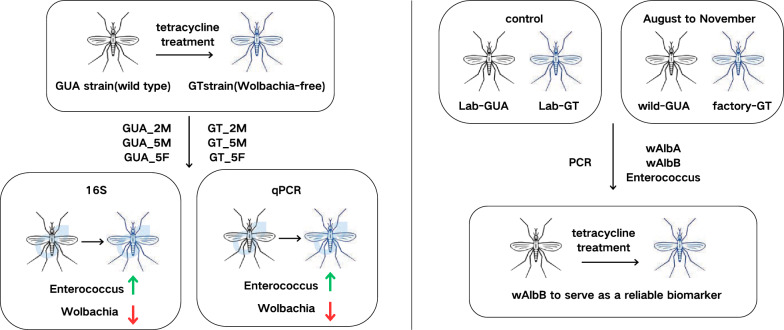

**Supplementary Information:**

The online version contains supplementary material available at 10.1186/s40249-025-01343-3.

## Background

Mosquito-borne diseases pose a serious public health threat due to their rapid spread and severe impact [1]. Vector control is the main preventive measure, given the lack of vaccines and treatments [2]. Traditional methods, like reducing breeding sites and spraying insecticides, are now limited by rising mosquito resistance and adverse effects on health, non-target species, and the environment [3]. This highlights the urgent need for alternative control strategies. The sterile insect technique (SIT) offers one solution, involving the mass release of radiation-sterilized male mosquitoes to suppress target populations by preventing reproduction [4]. To effectively distinguish sterile males from wild males is crucial to evaluate male qualities including dispersal ability, lifespan, mating competitiveness and maintain sufficient release ratio of sterile to wild males for efficient suppression. Generally, sterile males are marked with dyes or powders to differentiate them from wild males [5]. However, these methods are labor-intensive, have limited durability, and can negatively affect the fitness and competitiveness of sterile mosquitoes, thereby reducing program effectiveness [6]. Therefore, it is critical to find endogenous biomarkers for differentiation.

Insects host symbiotic microorganisms in specialized organs or cells, which support nutritional metabolism, reproduction, immune regulation and pathogen defense [7–9]. It is reported that over 10% of insect species have evolved specialized somatic cells, known as bacteriocytes, for harboring symbiotic bacteria, while others may reside in different host tissues or cells [10]. These bacteria, such as *Wolbachia pipiens*, *Rickettsia*, *Arsenophonus* and *Serratia marcescens*, play a crucial role in various physiological processes of their hosts. Female insects are capable of vertically transmitting these bacteria to their progeny, where they typically establish lifelong infections within the hosts [11], albeit with population levels that may vary over time [12]. This allows these bacteria to persist stably in insect populations over the long term, suggesting that they could serve as potential biomarkers [13]. For example, antibiotics can be used to eliminate intracellular bacteria from hosts, or embryo injections can introduce symbionts to establish distinguishable populations based on the infection status of target bacteria [14]. Meanwhile, however, the composition of *Aedes* mosquito microbiota is also shaped by environmental factors such as temperature, larval habitat, and diet [15–17], leading to the variability across populations, mainly causing by environmentally acquired bacteria.

*Aedes albopictus* is one of the primary vectors for dengue transmission and naturally harbors two *Wolbachia* strains, at a high prevalence worldwide [18, 19]. This bacterium is distributed throughout all developmental stages, suggesting its important role as a symbiont in this mosquito species [20]. While tetracycline can remove *Wolbachia* from this mosquito species and initially causes fitness costs in the first few generations, these negative effects tend to disappear over time, suggesting that *Ae. albopictus* can adapt to *Wolbachia* loss [6, 21, 22]. Similar results were previously observed in a long-term inbreeding of *Wolbachia*-free *Ae. albopictus* strain (GT strain, established from its counterpart strain, GUA strain, from Guangzhou by using tetracycline for curing its native *Wolbachia*), with similar adaptability, radiation sensitivity, and susceptibility to arboviruses to GUA strain [6], suggesting that GT strain is a viable option for the control of *Ae. albopictus* in frame of SIT (using irradiated GT males, instead of irradiated wild males, to control *Ae. albopictus* population). The adaptation of *Ae. albopictus* to *Wolbachia* absence is accompanied by changes in its microbial community and knowing these changes is crucial for understanding host-microbiota interactions. In addition, understanding the difference in the microbial community can also provide potential biomarkers, beyond *Wolbachia*, for distinguishing the sterile and wild male mosquitoes in frame of SIT, ensuring better monitoring of release success and mating behaviors, particularly in settings where traditional marking methods are limited.

In this study, employing *16S r*RNA gene sequencing, we aim to accurately investigate the impacts of *Wolbachia* removal on the microbial composition and diversity of *Ae. albopictus*. Subsequently, we endeavor to identify biomarkers other than *Wolbachia* for differentiating sterile and wild male mosquitoes within the framework of the SIT. Our findings will offer pivotal evidence for enhancing the efficacy of SIT programs in the field control of *Ae. albopictus* populations.

## Methods

### Experiment 1: Effects of *Wolbachia* removal on the microbial communities in *Ae. albopictus*

#### Mosquito strains, sample preparation and 16S rRNA gene sequencing

We used two strains of *Ae. albopictus* for 16S ribosomal RNA (*16S r*RNA) gene sequencing: the GUA strain from Guangzhou, China, which carries two types of *Wolbachia* (*w*AlbA and *w*AlbB), and the *Wolbachia*-free aposymbiotic GT strain. The GT strain was established from feeding GUA mosquitoes with tetracycline for five consecutive generations, followed by another two generations without tetracycline. The GUA and GT strains have been inbred in laboratory for 12 and 11 years, respectively. The mosquitoes were maintained with a 10% sugar solution at 27 ± 2 ℃ and 75 ± 10% humidity, with a 12-h light/dark cycle, according to standard rearing procedures [6, 21].

The *16S r*RNA gene sequencing was used to investigate the effects of *Wolbachia* removal on the microbial community of *Ae. albopictus* GUA mosquitoes. Samples from 6 groups (2 d-old GUA male adults (GUA_2M); 2 d-old GT male adults (GT_2M); 5 d-old GUA male adults (GUA_5M); 5 d-old GT male adults (GT_5M); 5 d-old GUA female adults (GUA_5F); 5 d-old GT female adults (GT_5F), were collected with an aspirator and then anesthetized by CO_2_. All these mosquitoes were provided with 10% sugar solution only. Following this, they were surface disinfected by dipping in 70% ethanol for 1 min, rinsed in sterile 1 × PBS (phosphate buffer saline), and then stored at -80 ℃ before DNA extraction. Each group contained five replicates, with each replicate comprising five adults.

DNA was extracted using the QIAamp Fast DNA Stool Mini Kit (Qiagen, Hilden, Germany) and then purified using the DNA Gel Extraction Kit (Axygen, California, USA), following the manufacturer’s protocols. The next generation sequencing (NGS) analysis targeted the *16S r*RNA gene at V3–V4 regions, which were amplified using the primers 338F (5’-ACTCCTACGGGAGGCAGCA-3’) and 806R (5’-GGACTACHVGGGTWTCTAAT-3’). Sequencing was performed using the Illumina MiSeq platform (Illumina, USA) and conducted by Majorbio Bio-Pharm.

### Bioinformatic analysis

Raw FASTQ files were de-multiplexed using an in-house perl script, and then quality-filtered by fastp version 0.19.6 [23] and merged by FLASH version 1.2.7 [24] with the following criteria: (i) the reads were truncated at any site receiving an average quality score of < 20 over a 50 bp sliding window, and the truncated reads shorter than 50 bp were discarded, reads containing ambiguous characters were also discarded; (ii) only overlapping sequences longer than 10 bp were assembled according to their overlapped sequence. The maximum mismatch ratio of overlap region is 0.2. Reads that could not be assembled were discarded; (iii) Samples were distinguished according to the barcode and primers, and the sequence direction was adjusted, exact barcode matching, 2 nucleotide mismatch in primer matching. Then the optimized sequences were clustered into operational taxonomic units (OTUs) using UPARSE 7.1 [25, 26] with 97% sequence similarity level. The most abundant sequence for each OTU was selected as a representative sequence. The taxonomy of each OTU representative sequence was analyzed by RDP Classifier version 2.2 [27] against the *16S r*RNA gene database (eg. Silva v138) using confidence threshold of 0.7. The metagenomic function was predicted by PICRUSt2 (Phylogenetic Investigation of Communities by Reconstruction of Unobserved States) [28] based on OTU representative sequences. PICRUSt2 is a software containing a series of tools as follows: HMMER was used to aligns OTU representative sequences with reference sequences. EPA-NG and Gappa were used to put OTU representative sequences into a reference tree. The castor was used to normalize the *16S r*RNA gene copies. MinPath was used to predict gene family profiles, and locate into the gene pathways. Entire analysis process was accord to protocols of PICRUSt2.

### Quantitative analysis of the major bacteria groups

Based on the *16S r*RNA sequencing results, four major genera were quantified by quantitative PCR (qPCR), *Asaia*, *Wolbachia*, *Enterococcus* and *Elizabethkingia*, among the above 30 sequenced samples. The *Ae. albopictus* ribosomal protein S6 (*rps6*) gene was chosen as a reference. The primers used for *Asaia*, *Wolbachia* and *Enterococcus* along with the annealing temperature (TA) and melting temperature (TM) were similar to those in previous studies [29, 30]. The primers for *Elizabethkingia* were designed as: SECY F1_4 (5’-GTTTTTACGTTCACGCTCATCTTGGT-3’) and SECY R2 (5’-AGTAAGCCTAAAAGCCCAGAAG-3’) [31]. The amplification was performed using SYBR Green Supermix (Vazyme, Nanjing, China). The reaction mixture (20 μl) consisted of 10 μl of 2 × Supermix, 2 μl DNA template, 0.4 μl paired primers (10 μmol/L) and 7.2 μl ddH_2_O. qPCR was performed with a LightCycler 96 real-time PCR detection system (Roche, Basel, Switzerland). An initial denaturation at 95 ℃ for 3 min was followed by 40 cycles consisting of denaturation at 95 ℃ for 15 s, at annealing temperature for 30 s, and extension at 72 ℃ for 30 s. The fluorescence collection was performed at the extension stage. To check and confirm the quality of amplification, a melting profile was generated for the amplicon over a temperature range of 65–95 ℃. Melting curves for each sample were analyzed after each run to check that there was no primer dimer formation. The relative abundance of bacteria was determined by using the 2^−ΔΔCt^ calculation method.

### Experiment 2: Biomarker validation between the sterile and the wild *Ae. albopictus* male mosquitoes

#### Wild mosquito collection and mass production of the sterile GT males

Based on the *16S r*RNA and qPCR results, the presence of *Wolbachia* and/or *Enterococcus* can be used to distinguish the GT and GUA strains. To distinguish the GT strain from wild (GUA) male *Ae. albopictus* mosquitoes, it is necessary to compare the presence of *Wolbachia* and *Enterococcus* between the radiation-sterilized GT and wild males. This comparison is essential for identifying reliable microbial markers that can differentiate sterile males from wild males in SIT programs. Therefore, wild *Ae. albopictus* male mosquitoes were monthly collected by human-baited collection from July to November, 2023 in the northern campus of Sun Yat-sen University, China (Latitude: 23°7′39.74’’N, Longitude: 113°17′22.07’’E). Briefly, well-protected volunteers stood in the selected 3 sample sites and used a locally manufactured hand-held electric aspirator to collect the adult mosquitoes flying around the performers for 15 min. Twelve male samples from the wild were randomly selected for the detection of *Wolbachia* (*w*AlbA and *w*AlbB) and *Enterococcus* via PCR.

The lab-reared GT strain was brought to mass rearing under factory conditions from October 2020. Mass production of GT males included adult and larval rearing was same as previously described [32]. Male mosquitoes were immobilized and packed in a cool room at 10 ℃. Then the male adults were exposed to X-ray at dose of 60 Gy, for achieving > 99.0% sterility [32]. Each month twelve factory-reared and irradiated GT male samples were randomly collected and detected for *Wolbachia* (*w*AlbA and *w*AlbB) and *Enterococcus* infection in parallel. Twelve male samples from the lab-reared GUA strain were used as positive controls, while twelve from the lab-reared GT strain were used as negative controls. All the lab-reared and factory-reared male mosquitoes were tested at 2 to 3 days old.

Due to environmental conditions might affect the diversity, abundance or composition of microbiota in male mosquitoes, it is necessary to validate the *Wolbachia* biomarker with the sterile males post released in the field. Two thousand irradiated GT males were marked with fluorescent powder (green) and then released in sample site 2 in May 2025. Following mosquitoes were collected by human-baited collection at 24 h, 48 h and 72 h and 96 h post release. The collected mosquitoes were killed and then identified into two groups under a stereo-microscope: the males with fluorescent powder were considered as the recaptured irradiated GT males while the unmarked samples were as wild males. Twelve male samples of each group captured at 48 h and 96 h post release were randomly for the detection of *Wolbachia* (*w*AlbA and wAlbB)*.* All the male samples were detected if the collected number was less than 12. The un-released irradiated GT males were used as control.

### DNA extraction and PCR amplification

DNA was individually extracted from the above-mentioned samples following the protocol of FastPure Cell/Tissue DNA Isolation Mini Kit (Vazyme, Nanjing, China). DNA was concentrated to > 15 ng/μl according to the original concentration estimated by a NanoDrop 3000 spectrophotometer (Thermo Fisher, Delaware, USA) and was used for PCR analysis. Primers for *w*AlbA, *w*AlbB and *Enterococcus* are shown in Supplementary Table 1. PCR reaction was carried out in 25 μl reaction volumes consisting of 12.5 μl 2 × Rapid Taq Master Mix, 1 μl primers (10 μmol/L) and 1 μl of DNA template. The PCR conditions followed for each step included 3 min at 95 ℃ for the initial denaturation step followed by 30 cycles of 15 s at 95 ℃, 15 s at 55/52 ℃, 5 s at 72 ℃ and 5 min at 72 ℃ for the final extension. PCR products were electrophoresed on a 1% agarose gel, which contained 1 μg/ml ethidium bromide. The DNA bands were visualized under UV light.

### Statistical analysis

Bioinformatic analysis of the *16S r*RNA sequencing data was carried out using the Majorbio Cloud platform (ps://cloud.majorbio.com). Based on the OTUs information, rarefaction curves and alpha diversity indices including observed OTUs, Chao1 richness, Shannon index and Good’s coverage were calculated with Mothur v1.30.1 [33]. The similarity among the microbial communities in different samples was determined by partial least-squares discriminant analysis (PLS-DA) and principal coordinate analysis (PCoA) based on Bray-curtis dissimilarity using Vegan v2.5-3 package. The PERMANOVA test was used to assess the percentage of variation explained by the treatment along with its statistical significance using Vegan v2.5-3 package. The linear discriminant analysis (LDA) effect size (LEfSe) [34] (http://huttenhower.sph.harvard.edu/LEfSe) was performed to identify the significantly abundant taxa (phylum to genera) of bacteria among the different groups (LDA score > 4, *P* < 0.05).

Other data and statistical analyses were conducted using GraphPad Prism 6.0 software. Alpha diversity indices (Shannon and Chao) were compared between groups using Student’s *t*-test. The Wilcoxon rank-sum test was used to assess statistical differences between two groups, and the Kruskal-Wallis H test was applied for comparisons among multiple groups. The two-tailed Mann-Whitney test was used to compare the relative abundance of *Asaia*, *Wolbachia*, *Enterococcus*, and *Elizabethkingia* between the GUA and GT strains. For the assessment of the *Wolbachia* and *Enterococcus* infection proportion, data was arcsine transformed before analysis. The two-tailed Wilcoxon matched-pairs signed rank test was used to compare the *w*AlbA and *w*AlbB infection proportion between the wild strain collected from different months, while the *Enterococcus* infection proportion was compared between the wild strain and the factory-reared GT strain.

## Results

### *Ae. albopictus* GT strain showed similar microbial diversity but different composition to GUA strain

**Fig. 1:**
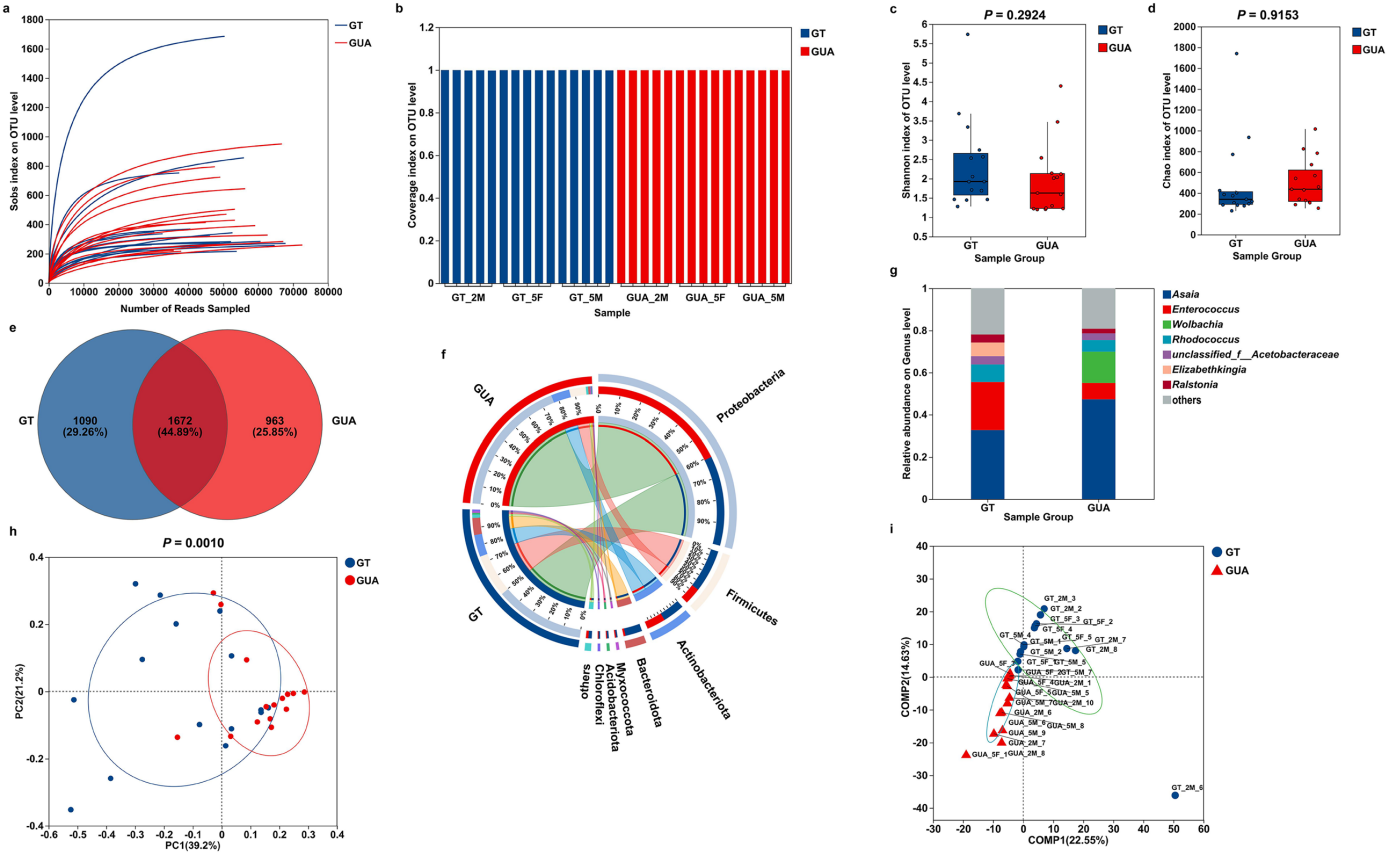
Microbial diversity and composition in GT and GUA strains of *Aedes albopictus*. **a** Rarefaction analysis of bacterial communities. **b** The good’s coverage of bacterial communities. **c** The boxplots of Shannon index; **d** The Chao index. The Shannon index and the Chao index were constructed to evaluate microbial diversity. **e** Venn diagram analysis of microbial communities at the OTU level. **f** CIRCOS plot showing the distribution of phylum between the GT and GUA strain mosquitoes. **g** Community bar chart at the genus level. **h** PCoA score plot of the bacteria in the GUA and GT strains on OTU level. **i** PLS-DA score plot of the bacteria in the GUA and GT strains on OTU level

Following size filtering, quality control, and chimera removal, a total of 1,575,628 high-quality sequences were obtained from 30 samples from two *Ae. albopictus* strains. Rarefaction analysis showed that the number of sequences increased sharply before reaching a plateau (Fig. 1a), indicating comprehensive coverage of bacterial diversity in these samples. Good’s coverage was 99.9% in all the sequencing samples (Fig. 1b), indicating sufficient sequencing depth. To evaluate alterations in microbial diversity between the GUA and GT strains, microbial alpha diversity was shown using Shannon and Chao diversity indices (Fig. 1c, d), with no significant differences observed (*P* = 0.2924 and *P* = 0.9153, respectively). In addition, 3653 OTUs were identified, including 45 phyla, 1048 genera, and 1953 species, which were annotated for subsequent analyses.

Venn diagram analysis based on the OTU abundance was performed to identify the common and unique OTUs between the GUA and GT strains (Fig. 1e). A total of 1672 OTUs shared by both strains, referred to as core microorganisms, were predominantly composed of Proteobacteria (64.27%), Firmicutes (16.09%), Actinobacteriota (11.22%) and Bacteroidota (4.96%) (Fig. 1f), accounting for over 96.00% of the total microbial in the two strains. The unique OTU number of the GUA strain was lower than that of the GT strain (Fig. 1e). The relative abundance for the top ten genera varied in the two strains. For example, *Asaia* was the most dominant genus in both strains (GUA: 47.33%, GT: 32.69%), though no significant difference observed (*P* = 0.1466) (Fig. 1g, Suppl Fig. 1a). Similarity, no significant difference was observed on *Rhodococcus* (*P* = 0.1150) (Suppl Fig. 1b), unclassified_f__*Acetobacteraceae* (*P* = 0.1710) (Suppl Fig. 1c), *Elizabethkingia* (*P* = 0.1250) (Suppl Fig. 1d), *Ralstonia* (*P* = 0.1150) (Suppl Fig. 1e), *Burkholderia-Caballeronia-Paraburkholderia* (*P* = 0.0740) (Suppl Fig. 1f) and *Pseudomonas* (*P* = 0.4800) (Suppl Fig. 1 g) in the two strains (Fig. 1g). However, significant difference was observed on *Pseudonocardia* (*P* = 0.0101) (Suppl Fig. 1 h), *Enterococcus* (*P* = 0.0062) (Suppl Fig. 1i) and *Wolbachia* (*P* = 0.0000) (Suppl Fig. 1j). *Wolbachia* was only observed in the GUA strain, while *Enterococcus* showed a higher relative abundance in GT strain compared to GUA strain (22.85 vs 7.75%) (Fig. 1g).

To further explore the microbial compositions between GUA and GT strains, we used PCoA (based on Weighted-Unifrac distances) at the OTU level. This analysis considered species relationships, composition abundance and evolutionary relationships, revealing clear segregation between the microbiota of the two strains (Fig. 1h, *P* = 0.0010). Additionally, Partial Least Squares Discriminant Analysis (PLS-DA) and the PERMANOVA analysis (Table 1) were conducted, showing distinct separation between the two strains (Fig. 1i). The two results from the beta-diversity analysis suggested that GUA and GT strains maintained different bacterial composition, and the differences might be due to the variation in the relative abundance of the major genera that were observed. (Fig. 1g). Our results suggested that the *Ae. albopictus* GT strain showed similar microbial diversity but different composition to GUA strain. Additionally, we conducted KEGG pathway prediction analysis on samples from the two strains and found no significant differences in their major metabolic pathways (Suppl Fig. 2).

**Table 1:** PERMANOVA table of results for strain and strain-age-sex combined factors for genera level abundance

Characteristics	SumsOfSqs	MeanSqs	F_Model	R2	*P*_value	*P*_adjust
GT & GUA	0.6414	0.6414	4.3240	0.1338	0.0010^**^	0.0010^**^
GT_5F & GUA_5F	0.1529	0.1529	1.4580	0.1542	0.1710	0.1710
GT_2M & GUA_2M	0.5981	0.5981	3.7515	0.3192	0.0090^**^	0.0090^**^
GT_5M & GUA_5M	0.5418	0.5418	4.8424	0.3771	0.0050^**^	0.0050^**^

Thirty samples were studied with six biological replicate each. Within the table, statistically significant difference (*P* < 0.05) were observed in bold value in strain factor and strain-age-sex combined factors

GUA_5F: 5 d-old GUA female adults; GT_5F: 5 d-old GT female adults; GUA_2M: 2 d-old GUA male adults; GT_2M: 2 d-old GT male adults; GUA_5M: 5 d-old GUA male adults; GT_5M: 5 d-old GT male adults

### GT females showed similar microbial diversity and composition to GUA females

**Fig. 2:**
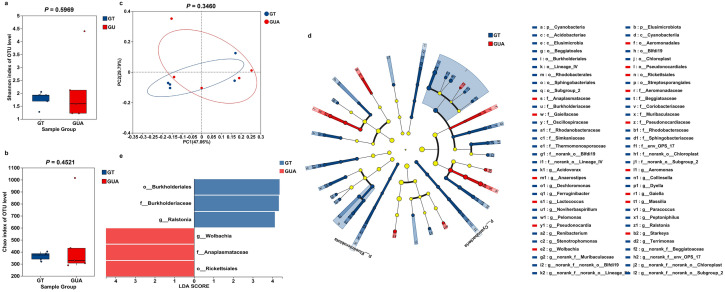
Microbial diversity and composition in GT and GUA female *Aedes albopictus* at age of 5-day old. **a** The boxplots of Shannon index. **b** The Chao index. The Shannon index and the Chao index were constructed to evaluate microbial diversity. **c** PCoA score plot of the bacteria on OTU level. **d** The LEfSe diagram of bacteria in GT and GUA strains. Red indicates significant enrichment in GUA, blue indicates significant enrichment in GT, and both indicate microbial taxa that have a significant impact on inter-group differences. Light yellow nodes indicate no significant difference or no significant impact on inter-group differences for microbial taxa. **e** Indicator bacteria with LDA scores of 4 or greater in bacterial communities in GT and GUA females. LDA score on the x-axis represents score changes in GT females compared to GUA females at the bottom left of the figure, showing the varying impacts of identified indicator species on the differences between GT and GUA females

To understand the microbial diversity and composition among the samples, the 30 samples were divided into six groups: GUA_2M, GUA_5M, GUA_5F, GT_2M, GT_5M and GT_5F. Regarding to female mosquitoes, no significant difference was observed on the microbial diversity between the GT and GUA strains based on Shannon (*P* = 0.5969) and Chao (*P* = 0.4521) indices (Fig. 2a, b). Additionally, similar bacterial composition was also observed in females of these two strains based on the PCoA analysis (Fig. 2c, P = 0.3460). The PERMANOVA analysis also indicated no significant differences between the two groups (Table 1).

Linear discriminant analysis effect size (LEfSe) method was further employed to identify the species/genus-specific difference in females. The GT females were characterized by a preponderance of *Ralstonia* (*Burkholderiales*), *Paracoccus* (*Rhodobacterales*)*,* and *Pelomonas* (*Sphingobacteriales*) (Fig. 2d) while *Wolbachia*, *Anaplasmataceae*, *Pseudonocardia* (*Rickettsiales*)*,* and *Massilia* (*Pseudonocardiales*) were more consistently present in the GUA females (Fig. 2e). Specifically, sequence analysis showed that the top five genera in the GUA females were *Asaia* (41.72%), *Enterococcus* (20.78%), *Wolbachia* (8.27%), *Rhodococcus* (2.44%) and *Ralstonia* (1.42%) (Suppl Fig. 3a) while the four predominant genera in the GT females were *Asaia* (55.08%), *Enterococcus* (19.22%), *Rhodococcus* (6.76%) and *Ralstonia* (3.63%) (Suppl Fig. 3b). Among of these genera bacteria, the presence of *Wolbachia* was the most differences between GT and GUA females.

### GT males showed similar microbial diversity but different composition to GUA males

**Fig. 3:**
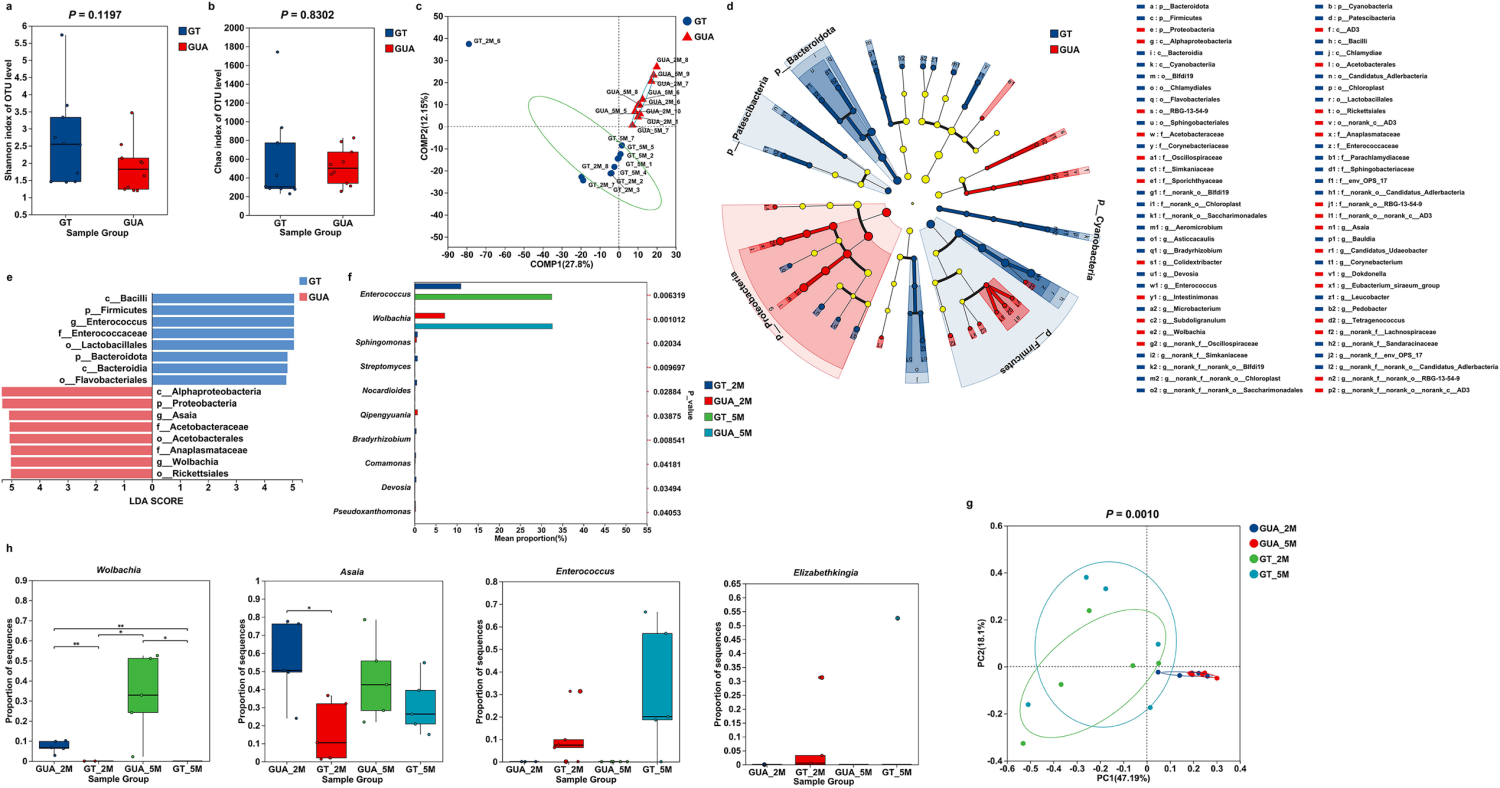
Microbial diversity and composition in GT and GUA male *Aedes albopictus* at age of 2-day and 5-day old. **a** The boxplots of Shannon index. **b** The Chao index. The Shannon index and the Chao index were constructed to evaluate microbiome diversity. **c** PLS-DA score plot of the bacteria in the GUA and GT strains on OTU level. **d** The LEfSe diagram of bacteria in GT and GUA strains. Red indicates significant enrichment in GUA, blue indicates significant enrichment in GT, and both indicate microbial taxa that have a significant impact on inter-group differences. Light yellow nodes indicate no significant difference or no significant impact on inter-group differences for microbial taxa. **e** Indicator bacteria with LDA scores of 4 or greater in bacterial communities in GT and GUA males. LDA score on the x-axis represents score changes in GT males compared to GUA males at the bottom left of the figure, showing the varying impacts of identified indicator species on the differences between GT and GUA males. **f** Kruskal-Wallis H test bar plot on genus level of GT and GUA males at 2 and 5 days old. **g** PCoA score plot of the bacteria in the four male groups on OTU level. **h** Kruskal-Wallis H test of *Wolbachia, Asaia, Enterococcus* and *Elizabethkingia* in GT and GUA males at 2 and 5 days old

In terms of male mosquitoes, no significant difference was observed on the microbial diversity based on Shannon (Fig. 3a, *P* = 0.1197) and Chao indices (Fig. 3b,* P *= 0.8302) between the GT and GUA strains. However, significant difference was observed on the composition based on the PLS-DA (Fig. 3c) and the PERMANOVA analysis (Table 1).

LEfSe was employed to identify the species/genus-specific difference in males. The GT males were characterized by a preponderance of *Bacilli*, *Enterococcus* (*Firmicutes*), *Enterococcaceae* and *Lactobacillales* (Fig. 3d, e), while *Alphaproteobacteria*, *Asaia* (*Proteobacteria*), *Acetobacteraceae* (*Acetobacterales*) and *Wolbachia* (*Anaplasmataceae*) were more consistently present in the GUA males (Fig. 3d, e). Specifically, sequence analysis showed that the abundance of the top four genera in GUA males were *Asaia*, *Wolbachia*, *Rhodococcus* and *Raistonia*, with *Wolbachia* being the second most abundant and *Enterococcus* nearly absent (Suppl Fig. 3c, d). In GT males, *Asaia*, *Enterococcus*, *Rhodococcus*, *Elizabethkingia* and *Ralstonia* were prominent, whereas *Enterococcus* being the second most abundant genus (Suppl Fig. 3e, f). Among of these genera bacteria, the presence of *Wolbachia* (*P* = 0.0010) or *Enterococcus* (*P* = 0.0063) showed significant statistical differences between GT and GUA males (Fig. 3f).

### Age significantly affected microbial composition in male mosquitoes

This study further revealed age-related differences in bacterial composition among the four male groups. The PCoA results have shown significant differences among the four groups (*P* = 0.0010) (Fig. 3g). In the GUA strain, the proportion of *Asaia* decreased slightly with age, while the proportion of *Wolbachia* had a substantial increase (Suppl Fig. 3c, d). Meanwhile, in the GT strain, the proportion of *Asaia* and *Enterococcus* had a considerable increase with age (Suppl Fig. 3e, f). In 2-day male group, the distribution of *Asaia* was highest in the GUA strain, whereas the bacterial composition in the GT strain remained relatively well-distributed among the top five genera (Suppl Fig. 3c, e). Additionally, the proportion of *Asaia* was consistently higher in the GUA strain compared to the GT strain (Suppl Fig. 3c–f). Additionally, significant difference in bacteria like *Wolbachia* and *Enterococcus* were observed between the 2-day male groups and 5-day male groups (*P* < 0.05) (Fig. 3f). Compared to the 5-day male group, the 2-day male group had a significantly higher prevalence of bacterial genera, such as *Sphingomonas* (*P* = 0.0203), *Streptomyces* (*P* = 0.0097), *Nocardioides* (*P* = 0.0288), and *Qipengyuania* (*P* = 0.0388) (Fig. 3f)*.*

We found significant differences in the abundance of *Wolbachia* (2-day male groups: *P* = 0.0060; 5-day male groups: *P* = 0.0249) and *Asaia* (*P* = 0.0151) bacteria between GT and GUA strains, with *Wolbachia* being eliminated and its abundance increasing with age in the GUA males, while the abundance of *Asaia* decreased in GT males (Fig. 3h). However, *Enterococcus* (2-day male groups: *P* = 0.1095; 5-day male groups: *P* = 0.06123) and *Elizabethkingia* (2-day male groups: *P* = 0.3136; 5-day male groups: *P* = 0.3738) showed no significant differences between GT and GUA males (Fig. 3h), but *Enterococcus* was identified as a biomarker in the LEfSe results (Fig. 3d, e).

### The relative abundance of *Asaia*, *Wolbachia*, *Enterocossus* and *Elizabethinga*

**Fig. 4:**
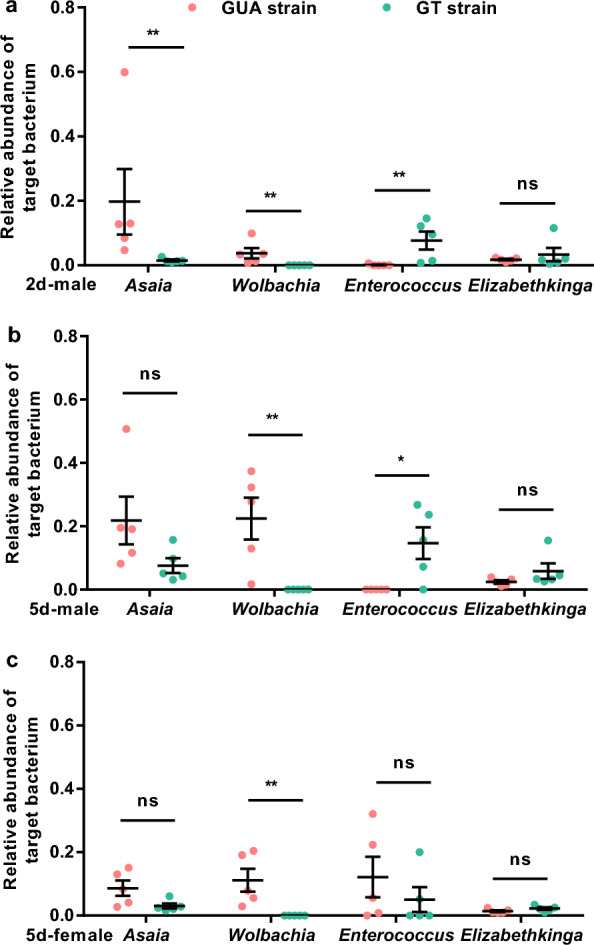
Relative abundance of *Asaia*, *Wolbachia*, *Enterococcus*, and *Elizabethkingia* in GUA and GT strains of *Aedes albopictus* mosquitoes. **a** Relative abundance of four genera in the 2-day-old male group. **b** Relative abundance of four genera in the 5-day-old male group. **c** Relative abundance of four genera in the 5-day-old female group

qPCR was further used to measure the relative density of 4 major bacterial genera. For *Asaia*, higher density was observed in GUA_2M group as compared to GT_2M group (Two-tailed Mann-Whitney *U*-test, *P* = 0.0079), while there was no difference in both 5-day male groups (GUA_5M vs GT_5M, *P* = 0.0556) and 5-day female groups (GUA_5F vs GT_5F, *P* = 0.0556) (Fig. 4a). GT males showed higher abundance of *Enterococcus* than GUA males, either at age of 2 days (*P* = 0.0079) or 5 days (*P* = 0.0476). However, this difference was not observed in 5-day female groups (*P* = 0.2381) (Fig. 4b). No significant difference was observed on *Elizabethkinga* between GUA and GT mosquitoes (*P* = 0.1508) (Fig. 4c). All GUA mosquito groups (GUA_2M, GUA_5M and GUA_5F) were positive with *Wolbachia* while the bacterium was not amplified in GT mosquitoes, confirming that *Wolbachia* was completely eliminated in the GT strain.

### *Wolbachia w*AlbB can serve as a biomarker to distinguish the sterile from wild male *Ae. albopictus*

**Fig. 5:**
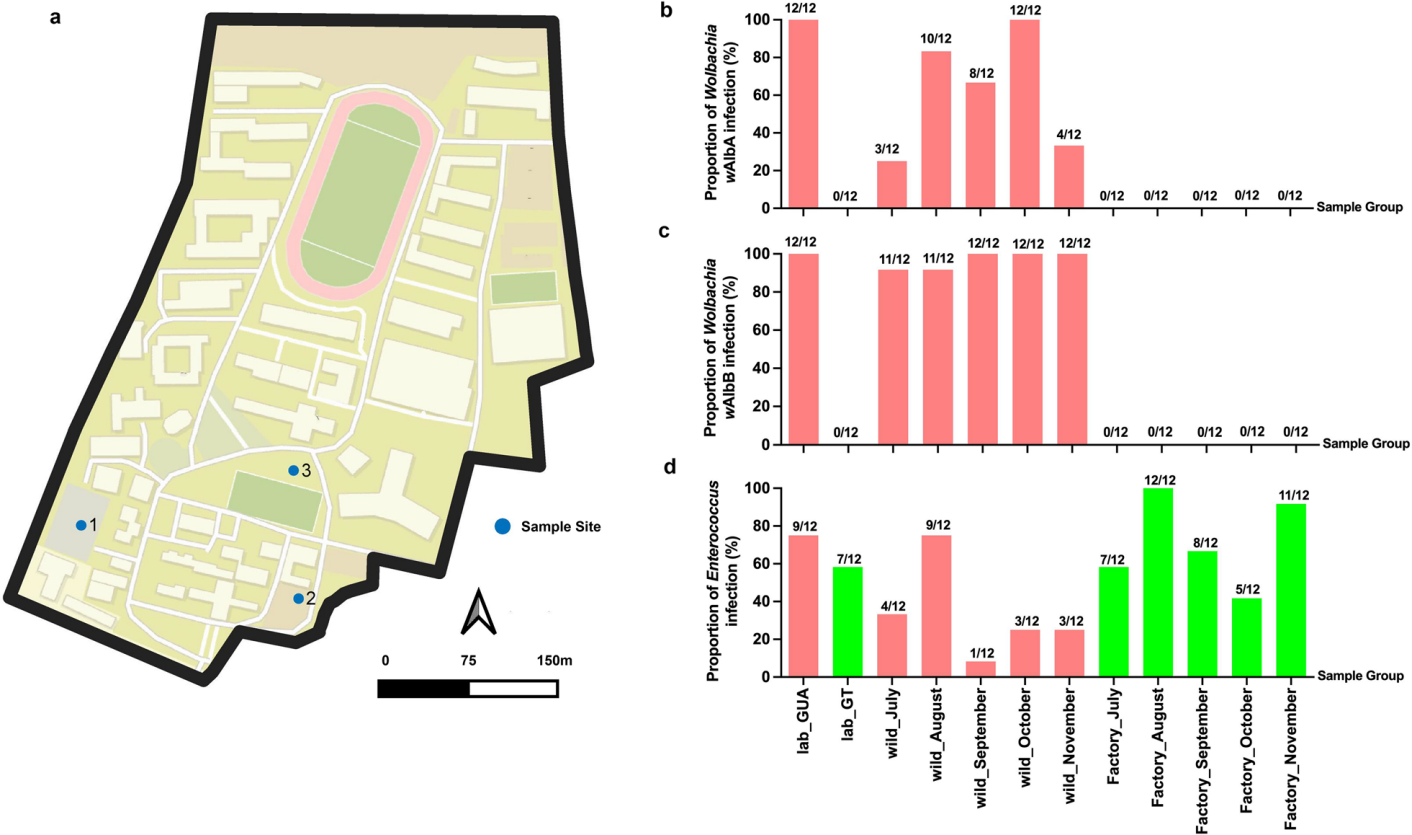
Proportion of *Wolbachia* and *Enterococcus* in male *Aedes albopictus*. **a** Spatial distribution of the monitoring method in Yuexiu District, Guangzhou City. Yellow points represent the positions to perform human-baited collection. N represents the North. **b** Proportion of *w*AlbA infection. **c** Proportion of *w*AlbB infection. **d** Proportion of *Enterococcus* infection. Within **a**, **b**, **c**, from left to right showed the laboratory strain GUA (lab-GUA), the laboratory strain GT (lab-GT), the wild strain collected from July to November 2024, and the mass-reared GT strain collected from July to November 2024

Three vegetation-rich sites in downtown Guangzhou, China, were selected for human-baited experiments to collect adult male *Ae. albopictus* mosquitoes (Fig. 5a). These sites included shrubbery near a parking lot, shrubbery near a basketball court, and shrubbery near a monument. The experiments were carried out monthly on the 15th day of each month, from July 2023 to November 2023, at all three sites. At each site, 30 male mosquitoes were collected, and 12 were selected for DNA extraction and PCR identification experiments. Male mosquitoes infected with both *Wolbachia* strains (*w*AlbA & *w*AlbB) were exclusively found, but in different proportions, in both the lab-GUA and the wild strains (Fig. 5b, c). The lab-GUA strain were 100% (12/12) double-infections with *w*AlbA and *w*AlbB. In terms of the wild strain, the *w*AlbB exhibited a mean of 96.7% (± 4.6%) (58/60) infection proportion and 61.7% (± 32.1%) (37/60) for *w*AlbA, though the difference was not statistically significant (Wilcoxon matched-pairs signed rank test, *P* = 0.1250) due to high variation observed in *w*AlbA. Conversely, both the lab-GT (0/12) and the mass-reared GT (0/60) strains were negative with *Wolbachia* in both* w*AlbA and *w*AlbB. All four tested strains were positive for *Enterococcus* (Fig. 5d). No significant difference was observed on the infection proportion of *Enterococcus* between the mass-reared and the wild strains (Wilcoxon matched-pairs signed rank test, *P* = 0.0625).

**Fig. 6:**
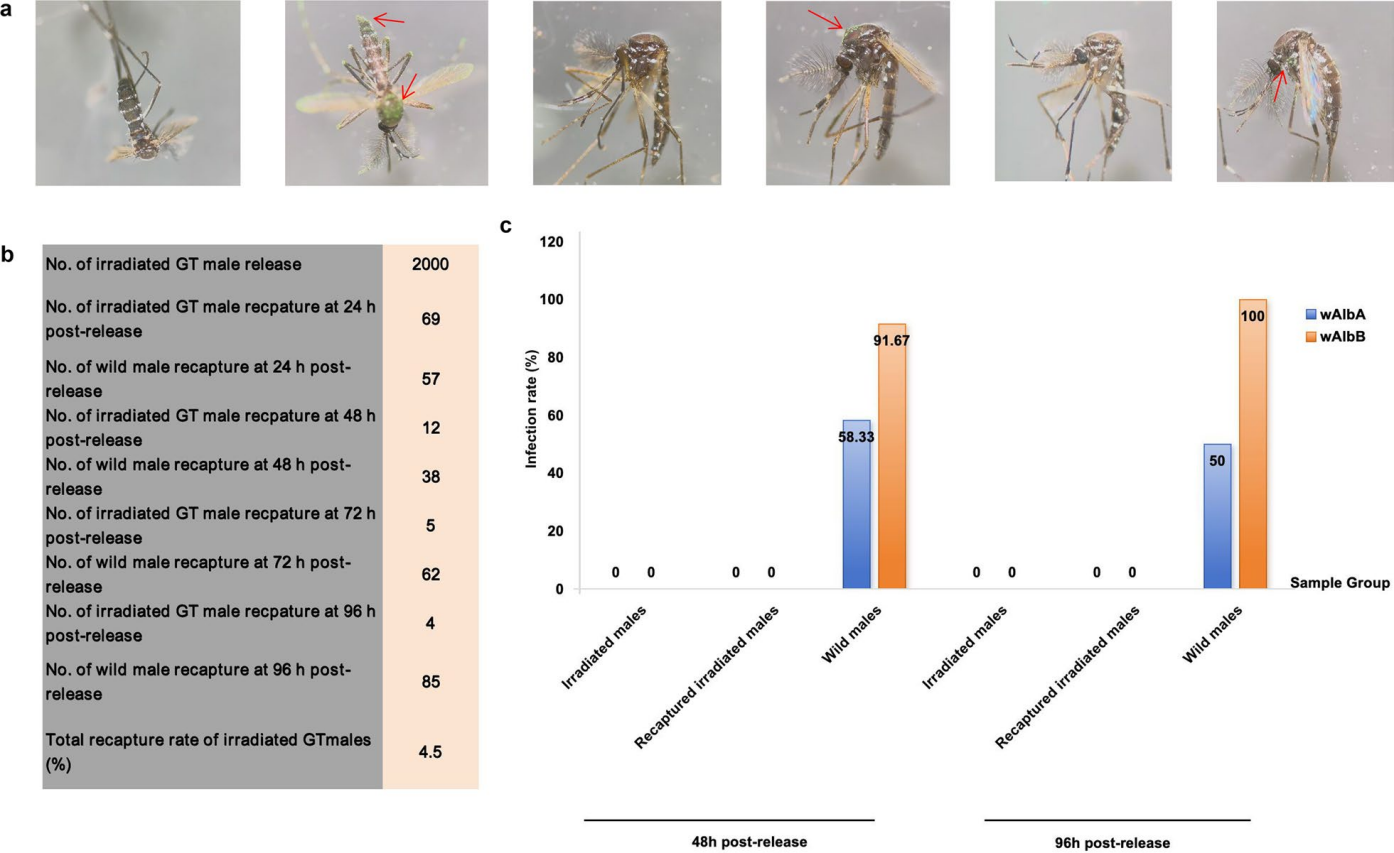
Validation of *w*AlbB as a reliable biological marker in the mark-release-recapture experiment. **a** Differentiation of male *Aedes albopictus* mosquitoes under a stereomicroscope to determine their origin (mass-reared or wild). From left to right: unmarked irradiated male, fluorescent marked irradiated male before release, wild male captured at 48 h, fluorescent marked irradiated male recaptured at 48 h post release, wild male captured at 96 h, and fluorescent marked irradiated male recaptured at 96 h post release. Fluorescent powder were indicated by the red arrows. **b** Numbers of marked, released, and recaptured male *Aedes albopictus*, along with the recapture rate of sterile males. **c** Proportion of *w*AlbA (blue) and *w*AlbB (orange) infections among the tested males

A total number of 90 irradiated GT males were recaptured with obvious fluorescent powder observed in these males, corresponding to a 4.5% (90/2000) recaptured rate within 4 days (Fig. 6a, b). The number of daily captured male mosquitoes with or without marking was shown in Fig. 6b. PCR results showed that all the marked male mosquitoes were negative with both *w*AlbA and *w*AlbB, either collected at 48 h (0/12) or 96 h post release (0/4). Similarity, all the irradiated GT males were negative with both *w*AlbA and *w*AlbB (0/24). Whereas, infection rates of 95.8% (23/24) for *w*AlbB and 54.2% (13/24) for *w*AlbA were respectively observed on the unmarked male mosquitoes, which were considered as wild males (Fig. 6c). Our results suggest that *Wolbachia w*AlbB is more stable than *w*AlbA recommended to be a suitable biomarker for distinguishing the sterile from wild male mosquitoes.

## Discussion

One of the key challenges in mosquito SIT is distinguishing the sterile mosquitoes from wild ones. We previously proposed that the symbiotic bacteria of mosquitoes, such as *Wolbachia*, could serve as a biomarker [32, 35]. By providing the *Ae. albopictus* GUA strain to continuous tetracycline treatment over multiple generations, we established the *Wolbachia*-free GT strain, which exhibits similar biological characteristics to the GUA strain [6]. This study further reveals that there are no significant differences in the microbial diversity between the GT and GUA mosquito strains. For male mosquitoes, age and strain are two significant factors influencing microbial composition, with the presence of *Wolbachia* or *Enterococcus* being the primary distinctions between GT and GUA males. Importantly, compared to *Enterococcus* or *Wolbachia w*AlbA, *w*AlbB exhibits higher infection rate in wild males (> 96.7%), suggesting that it may be a more suitable biological marker for distinguishing in SIT programs targeting *Ae. albopictus*.

Insects harbor diverse microbes that assist the host in performing essential functions [36]. One type is endosymbiotic bacteria, which establish a one-to-one (or one-to-few) relationship with the host, residing within specialized host cells and functionally complementing the host in various ways [37]. Another type is ectosymbiotic bacteria, which typically form a many-to-one relationship with the host, living in different host organs. The quantity and diversity of these bacteria can be easily influenced by environmental factors, leading to diversity or composition changes [7, 9, 38–41]. Normally the endosymbiotic bacteria has higher stability than ectosymbiotic bacteria in the same host. *Wolbachia* is an endosymbiotic bacterium primarily colonizing the host's reproductive system [42], capable of regulating various reproductive functions in the host, including cytoplasmic incompatibility (CI) [43]. Establishing a host strain without *Wolbachia* infection, using antibiotics such as tetracycline, doxycycline, or oxytetracycline, is a key model for studying *Wolbachia*-host interactions. We observed that the removal of *Wolbachia* resulting in the emergence or abundance changes of other bacteria (such as *Elizabethkingia*, *Enterococcus*, *Nocardia* and *Asaia*) in GT strain, though it showed no significant difference in microbial diversity to GUA strain based on the Shanno and Chao indices. The core mircobiota of both GT and GUA strains is predominantly composed of Proteobacteria (64.27%), Firmicutes (16.09%), Actinobacteria (11.22%), and Bacteroidetes (4.96%). Both strains maintained similar microbial diversity at the phylum level, with the core bacteria constituting over 96% of the microbial community, consistent with previous studies [14, 44, 45]. Further analysis revealed no significant differences in microbial diversity between the GT and GUA male mosquitoes, although significant differences in composition were observed. In contrast, no differences in diversity or composition were found in female mosquitoes (5 days old, sugar feeding). This disparity may relate to our analysis being limited to 5-day-old females, lacking comparisons for age and dietary factors. Previous study found that *Wolbachia*-free *Anopheles stephensi* female mosquitoes (LBT) showed similar diversity and composition of gut bacteria to the *Wolbachia*-infected strain (LB1), when mosquitoes were fed on sugar (7 days old). However, significant differences on composition emerged after blood feeding (14 days old), indicating that both the age and feeding regime factors reshape the gut bacteria [46].

According to the *16S r*RNA sequencing and qPCR results, *Enterococcus* was only observed in the GT male mosquitoes. However, the PCR results suggest that this bacterium can also exist in the lab and wild-collected *Ae. albopictus* populations, which naturally infected with two strains of *Wolbachia*. *Enterococcus* is widely distributed in nature and can be horizontally transmitted, suggesting that the differences in detection results may be related to the different batches of mosquitoes used in the experiments. Notably, both *16S r*RNA sequencing and qPCR results showed that GUA female mosquitoes infected with *Enterococcus*, consistent with our earlier findings in both male and female adult mosquitoes of GUA strain [30]. Additionally, the accuracy and sensitivity of detection methods may contribute to these discrepancies, as Wittmeier observed such differences when using different methods to detect *Enterococcus* [47]. Therefore, this suggests that *Enterococcus* cannot be relied upon as a reliable biological marker to distinguish the sterile from the wild male *Ae. albopictus*. There is no doubt that markers identified in lab conditions cannot be used directly as a reliable biomarker under field conditions as mosquito microbiota is significantly shaped by environmental factors [12–14]. These variables can greatly alter microbial community composition, particularly for environmentally acquired bacteria like *Asaia* and *Enterococcus*. In contrast, *Wolbachia* infection exhibited consistency, with GT populations, whether laboratory-reared or factory-reared, testing negative for *Wolbachia*, while both the lab and wild-collected populations were found to be super-infected with *w*AlbA and *w*AlbB, with *w*AlbB exhibiting higher stability infection. This indicates that *w*AlbB may serve as a suitable distinguishing marker. However, the prevalence of *w*AlbB in wild *Ae. albopictus* males are not 100% (mean = 96.7%), therefore, relying solely on the absence of *w*AlbB as a biomarker to identify irradiated males may yield false positives.

Ecological competition was found between *Asaia* and *Wolbachia*, particularly among *Anopheles* genus mosquitoes, where both can exhibit mutual exclusion when establishing a symbiotic relationship with the host [35, 45]. In terms of *Aedes* mosquitoes, Nejad found that all the wild *Ae. aegypti* mosquitoes were negative with *Wolbachia* infection but positive with *Asaia;* however, co-infection with *Wolbachia* and *Asaia* was observed in *Ae. albopictus*, where competition mostly observed in the reproductive systems, but not in the guts. Similar results were observed in the guts of *An. stephensi* LB1 or LBT females (7–15 days old), where *Asaia* constituted 5–8% of the total microbial, suggesting that the presence of *Wolbachia* does not affect the abundance of *Asaia*. On the contrary, we found that in the abundance of *Asaia* in GT mosquitoes, whether male or female, decreased following the removal of *Wolbachia*. Additionally, a comparison of 2-day-old and 5-day-old GUA males showed an increase in *Wolbachia* levels alongside a rise in *Asaia*, indicating that aside from competition, there may also be a mutualistic relationship between *Asaia* and *Wolbachia*. This complexity may be related to the use of young mosquitoes (2 and 5 days old) in this study, suggesting that the complex interactions between these two bacteria are involved.

The two *Ae. albopictus* strains (GUA and GT) used in this study have been maintained under laboratory conditions for over a decade. Although our previous researches have shown limited fitness costs between these strains [6, 21], their genetic differences, which have been linked to variations in mosquito fitness [48], may represent an additional factor contributing to the observed divergence in microbiota composition. Further investigations employing whole-genome sequencing approaches are required to comprehensively characterize the genetic distinctions between the GUA and GT strains and clarify their potential role in shaping strain-specific microbial communities.

## Conclusions

This study reveals that removal of *Wolbachia* from *Ae. albopictus* does not alter overall microbial diversity but significantly reshapes the bacterial composition, especially in males. Importantly, the endosymbiont *Wolbachia w*AlbB was stably present in wild-type males but absent in sterile males, supporting its application as a biomarker for distinguishing between the two in SIT programs. Although *Ae. aegypti* and most *Anopheles* mosquitoes do not carry this bacterium naturally, this approach can be adapted to identify specific endosymbiotic bacteria and cure them through antibiotic treatment, thereby establishing a usable biomarker for differentiation. In summary, this study provides new insights from a microbiological perspective for distinctions in frame of any mosquito SIT program.

## Supplementary Information

Additional file 1. Fig. 1 Comparative analysis of high-abundance microbes in different strains of *Aedes albopictus *by Wilcoxon rank-sum test on Genus level. (a) Asaia (*P* = 0.1466). (b) *Rhodococcus* (*P* = 0.1150). (c) Unclassified_f__*Acetobacteraceae *(*P* = 0.1710). (d) *Elizabethkingia *(*P* = 0.1250). (e) *Ralstonia *(*P* = 0.1150). (f) *Burkholderia-Caballeronia-Paraburkholderia *(*P* = 0.0740). (g) *Pseudomonas *(*P* = 0.4800). (h) *Pseudonocardia *(*P* = 0.0101). (i) *Enterococcus *(*P* = 0.0062). (j) *Wolbachia *(*P* = 0.0000).

Additional file 2. Fig. 2 Heatmap analysis of enrichment pathway in GT and GUA strains of *Aedes albopictus*. Using KEGG functional abundance statistics to predict the differences in metabolic pathways of *Ae. albopictus* in GT and GUA strains, with similar distribution of the main dominant functions among the samples was observed, indicating that the removal of *Wolbachia* does not significantly affect the primary metabolic pathways in *Ae. albopictus*.

Additional file 3. Fig. 3 Microbial composition of *Aedes albopictus* in six diverse groups of two strains (genus level). (a) Proportion of bacteria in GUA_5F group. (b) Proportion of bacteria in GT_5F group. (c) Proportion of bacteria in GUA_2M group. (d) Proportion of bacteria in GUA_5M group. (e) Proportion of bacteria in GT_2M group. (f) Proportion of bacteria in GT_5M group.

Additional file 4. Table 1 Primer sequence and amplification information.

## Data Availability

The datasets used and analyzed during the current study are available from the corresponding author on reasonable request.

## Abbreviations

*16S** r*RNA: 16S Ribosomal RNA
*rps6*: Ribosomal protein S6
LEfSe: Linear discriminant analysis effect size
OTUs: Operational taxonomic units
PCoA: Principal coordinate analysis
PLS-DA: Partial least-squares discriminant analysis
SIT: Sterile insect technique
GUA_2M: 2 d-old GUA male adults
GUA_5M: 5 d-old GUA male adults
GUA_5F: 5 d-old GUA female adults
GT_2M: 2 d-old GT male adults
GT_5M: 5 d-old GT male adults
GT_5F: 5 d-old GT female adults
