# Supplementary figures and images for: Effects of Wolbachia removal on microbial composition and diversity in Aedes albopictus: implication of using wAlbB for discriminating irradiation-based sterile and wild males
Source: Infect Dis Poverty. 2025 Jul 14;14:67. doi: 10.1186/s40249-025-01343-3 (PMC12257766; doi:10.1186/s40249-025-01343-3)

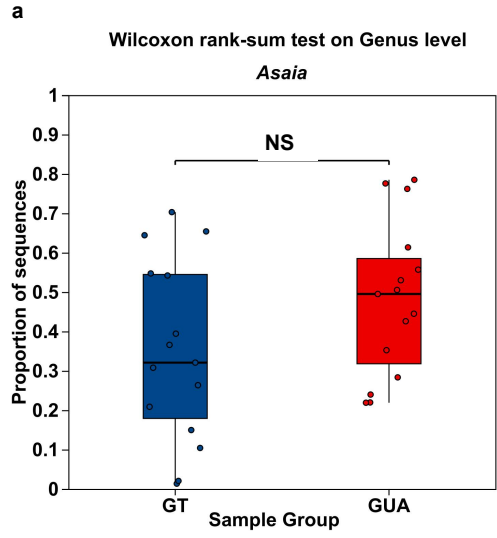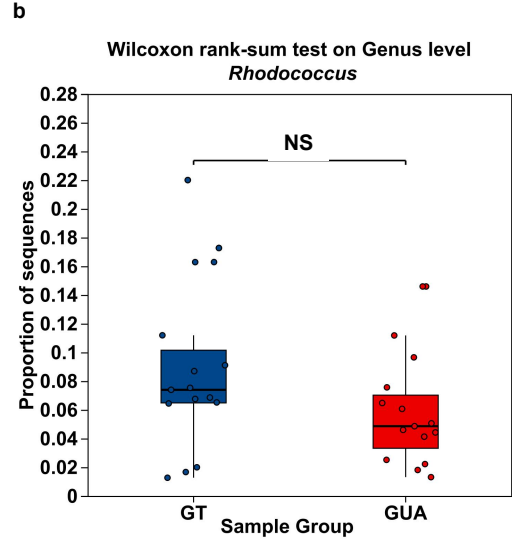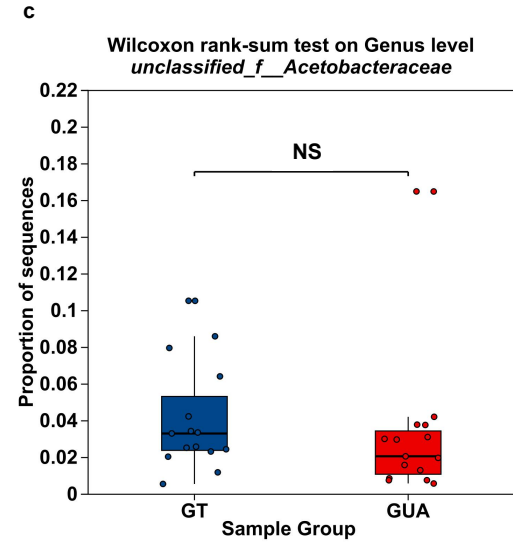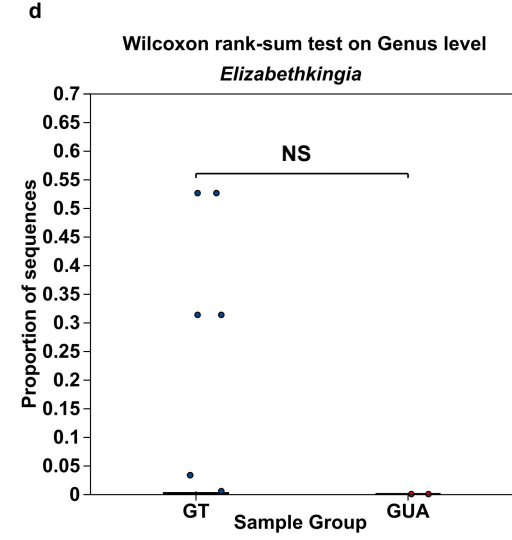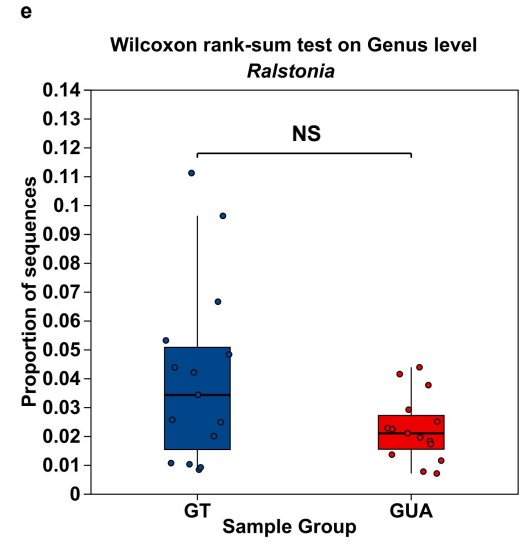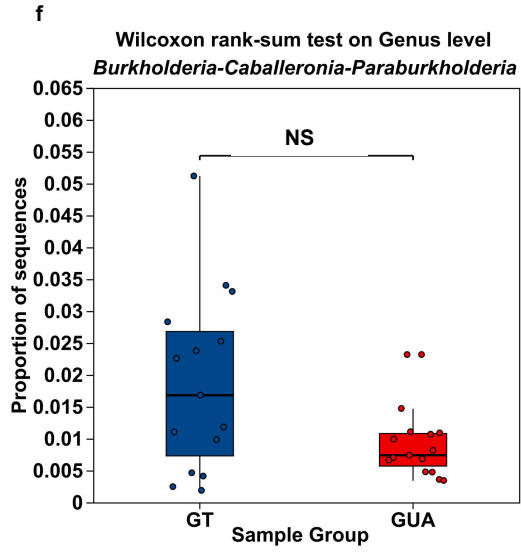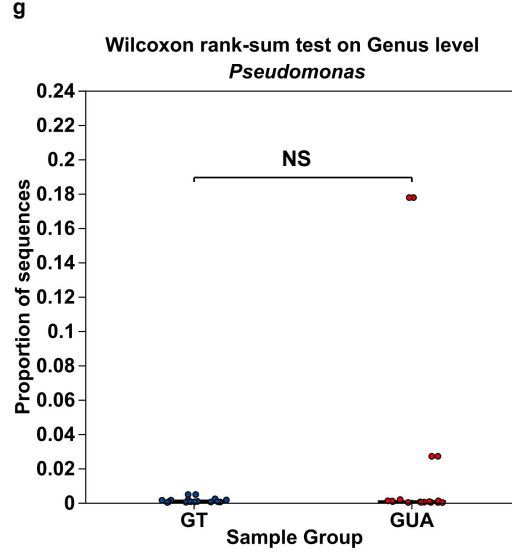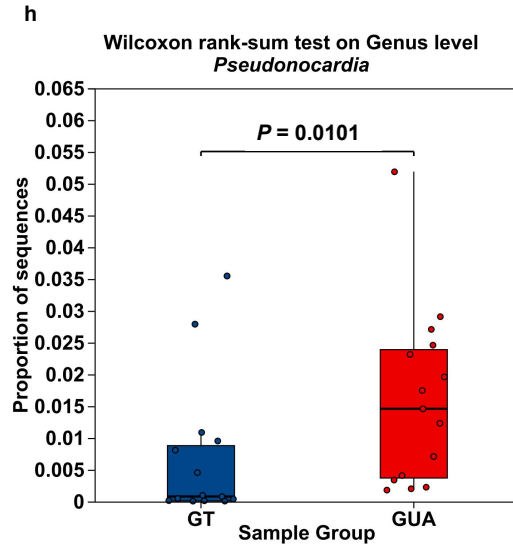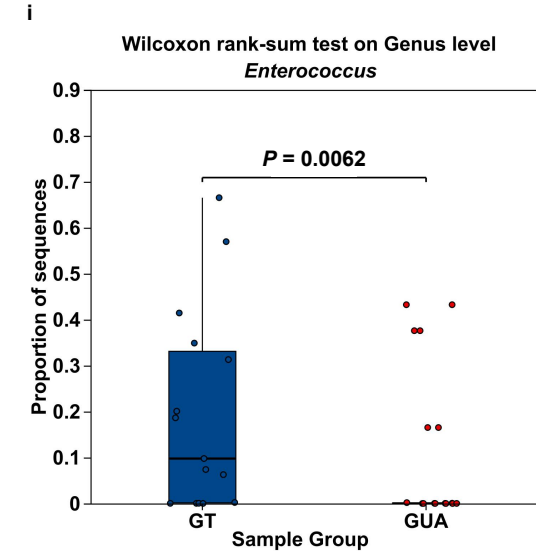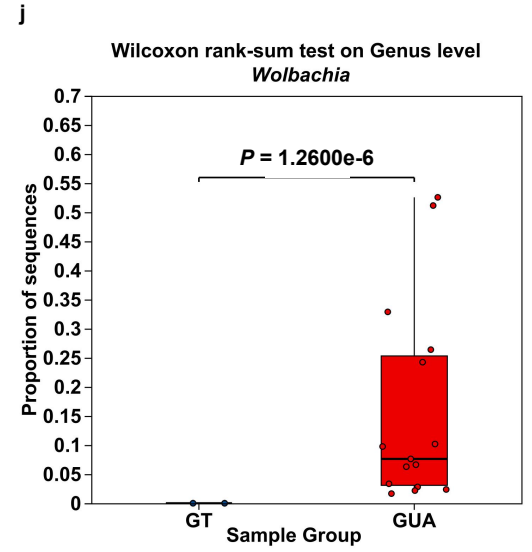

Supplement: Supplementary file 1 — Additional file 1. Fig. 1 Comparative analysis of high-abundance microbes in different strains of Aedes albopictus by Wilcoxon rank-sum test on Genus level. (a) Asaia (P = 0.1466). (b) Rhodococcus (P = 0.1150). (c) Unclassified_f__Acetobacteraceae (P = 0.1710). (d) Elizabethkingia (P = 0.1250). (e) Ralstonia (P = 0.1150). (f) Burkholderia-Caballeronia-Paraburkholderia (P = 0.0740). (g) Pseudomonas (P = 0.4800). (h) Pseudonocardia (P = 0.0101). (i) Enterococcus (P = 0.0062). (j) Wolbachia (P = 0.0000). [file 40249_2025_1343_MOESM1_ESM.pdf]

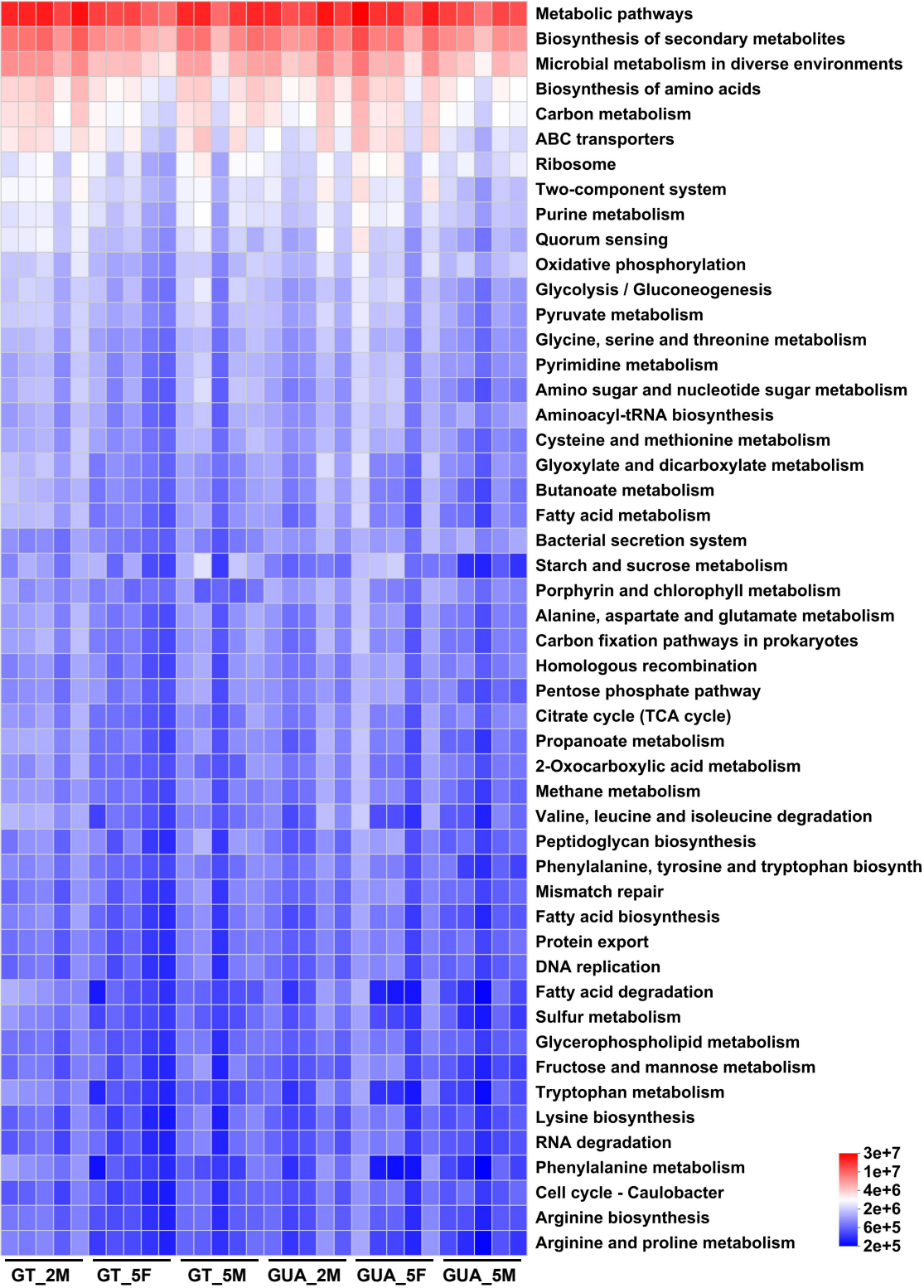

Supplement: Supplementary file 2 — Additional file 2. Fig. 2 Heatmap analysis of enrichment pathway in GT and GUA strains of Aedes albopictus. Using KEGG functional abundance statistics to predict the differences in metabolic pathways of Ae. albopictus in GT and GUA strains, with similar distribution of the main dominant functions among the samples was observed, indicating that the removal of Wolbachia does not significantly affect the primary metabolic pathways in Ae. albopictus. [file 40249_2025_1343_MOESM2_ESM.pdf]

a

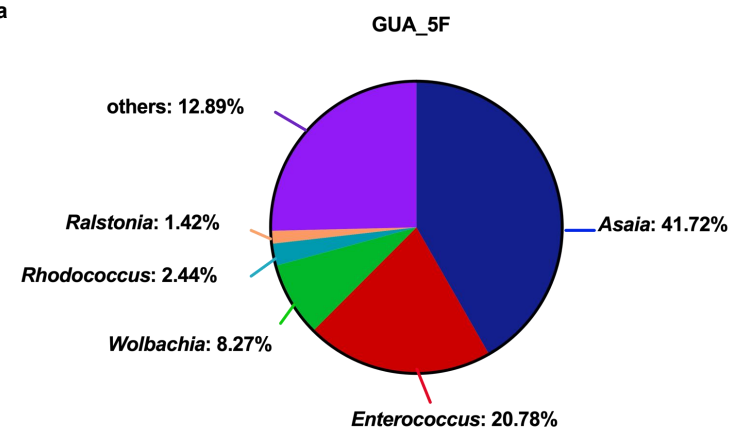

b

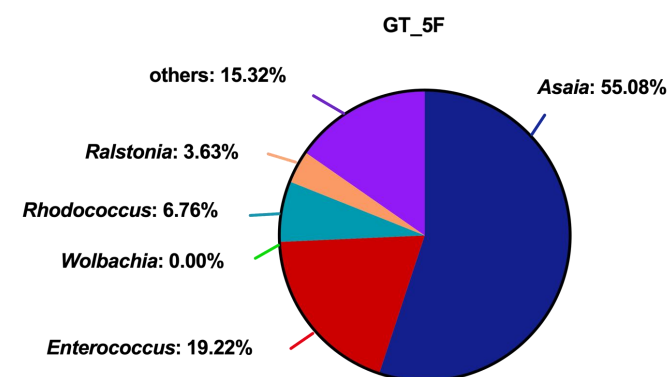

c

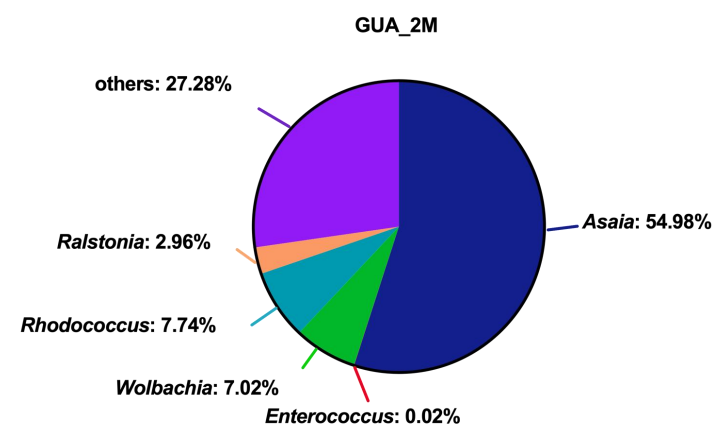

d

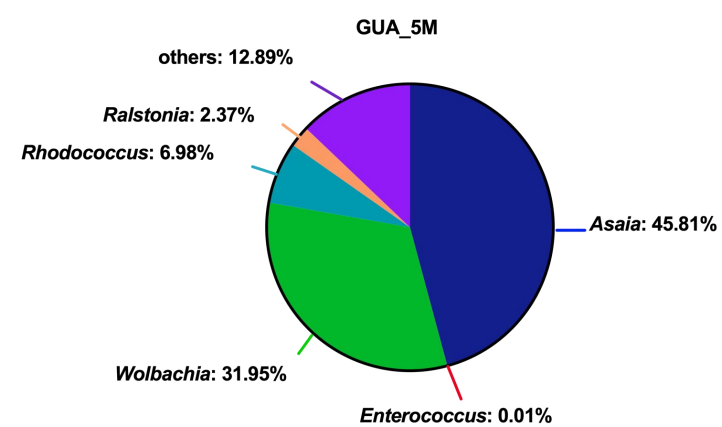

e

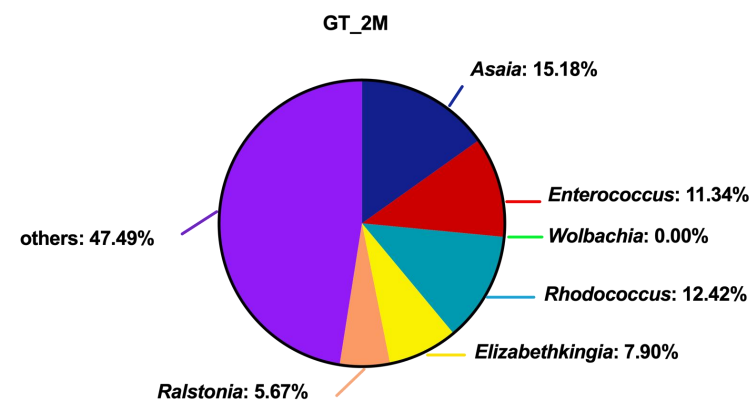

f

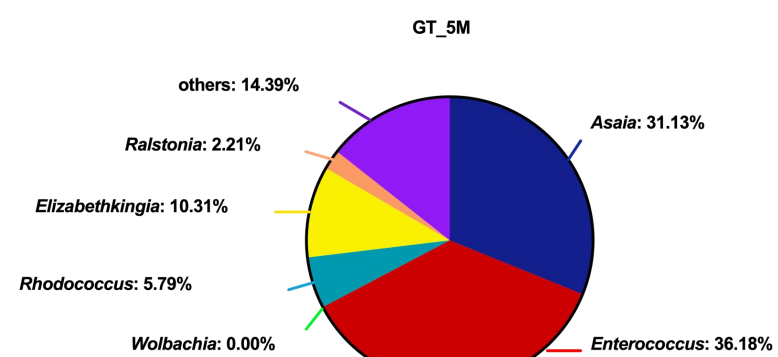

Supplement: Supplementary file 3 — Additional file 3. Fig. 3 Microbial composition of Aedes albopictus in six diverse groups of two strains (genus level). (a) Proportion of bacteria in GUA_5F group. (b) Proportion of bacteria in GT_5F group. (c) Proportion of bacteria in GUA_2M group. (d) Proportion of bacteria in GUA_5M group. (e) Proportion of bacteria in GT_2M group. (f) Proportion of bacteria in GT_5M group. [file 40249_2025_1343_MOESM3_ESM.pdf]
