# Supplementary material for: Effects of Wolbachia removal on microbial composition and diversity in Aedes albopictus: implication of using wAlbB for discriminating irradiation-based sterile and wild males
Source: Infect Dis Poverty. 2025 Jul 14;14:67. doi: 10.1186/s40249-025-01343-3 (PMC12257766; doi:10.1186/s40249-025-01343-3)
Supplement: Supplementary file 4 — Additional file 4. Table 1 Primer sequence and amplification information. [file 40249_2025_1343_MOESM4_ESM.pdf]

Supplementary Table 1. Primer sequence and amplification information

Organism	Primers	Primer sequences (5′-3′)	Annealing temperature (°C)	References
Asaia	Asa-For	GCGCGTAGGCGGTTTACAC	60	[44]
	Asa-Rev	AGCGTCAGTAATGAGCCAGGTT		
Elizabethkingia	SECY F1_4	GTTTTTACGTTCACGCTCATCTTGGT	64	[25]
	SECY R2	AGTAAGCCTAAAAGCCCAGAAG		
Enterococcus	Entbes-3	TTGCTAGAGTGCCCAACTGAAT	66	[23]
	Entbes-5	CCCTTACCAGGTCTTGACATCC		
*w*AlbA	328 F	CCAGCAGATACTATTGCG	55	[12]
	691R	AAAAATTAAACGCTACTCCA		
*w*AlbB	183 F	AAGGAACCGAAGTTCATG	55	[12]
	691R	AAAAATTAAACGCTACTCCA		
